# Elective implant removal in symptomatic patients after internal fixation of proximal humerus fractures improves clinical outcome

**DOI:** 10.1186/s12891-016-0977-z

**Published:** 2016-03-10

**Authors:** Yves P. Acklin, Christian Michelitsch, Christoph Sommer

**Affiliations:** Kantonsspital Graubünden, Loestr. 170, CH-7000 Chur, Switzerland

**Keywords:** Philos plate, Implant removal, Outcome

## Abstract

**Background:**

Operative treatment is the standard for severely displaced proximal humerus fractures, but functional impairment can persist. Retaining of the implant can be a reason and in other fracture situations has proved to ameliorate patient satisfaction. The aim of this study was to analyse the functional outcome after locking plate removal in proximal humerus fractures.

**Methods:**

In a two-year period, all symptomatic patients with plate osteosynthesis for proximal humerus fracture and hardware removal were retrospectively evaluated clinically and radiologically pre- and post-implant removal. Evaluation included Constant score, height of plate position and possible impingement, as well as intraoperative complications.

**Results:**

Twenty patients met the inclusion criteria. The mean age was 56 ± 12 years. The plates were placed 6.9 ± 3 mm distal to the greater tubercle. The operation was performed in 35 ± 10 min and no intraoperative complications were reported. The Constant score improved significantly after implant removal from 71 to 76 (*p* = 0.008).

**Conclusion:**

Symptomatic patients after locked plate osteosynthesis for proximal humerus fractures showed statistically significant improvement of the Constant score after implant removal.

## Background

The proximal humerus is one of the most frequent fractures [1, 2]. Marginally displaces fractures are treated conservatively, while in severely displaced fractures, operative therapy is preferred. Improved angular stable implants offer a predictable outcome in surgery. But all treatment options require long rehabilitation, with often persisting reduced range of motion and discomfort [3, 4]. After plate fixation, implant related complications e.g. impingement and meteorosensitivity can influence these clinical outcome parameters. It is therefore not surprising, that implant removal contributes to up to 30 % of all elective orthopaedic procedures [5]. But clinical amelioration after plate removal is rarely investigated [5, 6].

The purpose of this study is to determine, if implant removal can improve clinical outcomes in proximal humerus fractures.

## Methods

### Patient population

All patients undergoing an elective removal of a locking plate after minimal invasive plate osteosynthesis following a proximal humerus fracture were included in the retrospective study. The Cantonal Ethic Committee of Zurich approved the study (KEK-ZH-Nr. 2010-0421/4).

Patient age younger than 18 years were excluded. In addition, patients with existing disorders such as hemiplegia or other relevant neurologic disorders, non-union, primary or secondary intra-articular screw perforation, implant breakage, infection or avascular necrosis of the humeral head, polytrauma with an Injury Severity Score greater than 16, and patients with posttraumatic brachial plexus injury or peripheral nerve palsy were excluded.

**Fig. 1:**
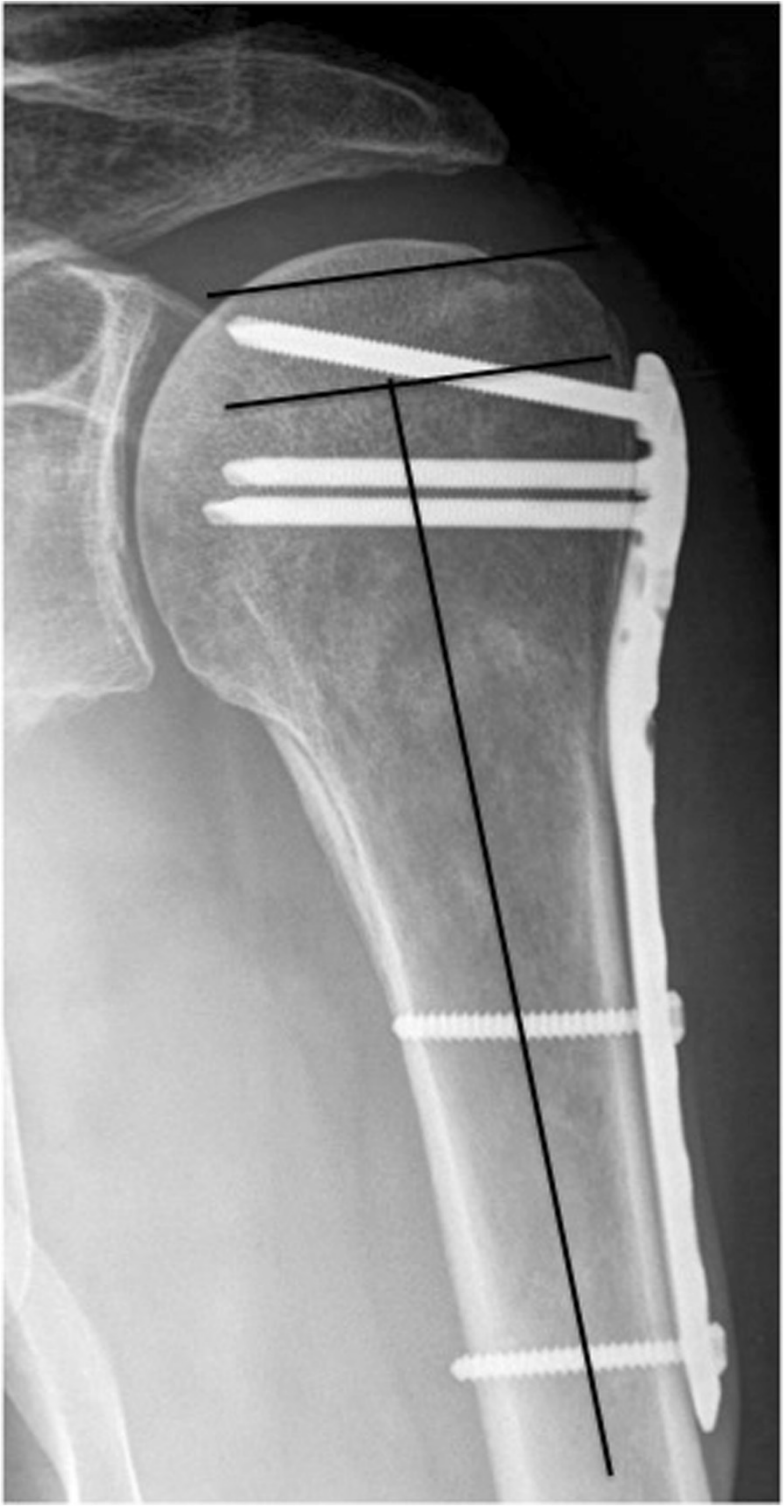
Plate height measurement according to the operation instructions

Data collection included patients’ age, sex, associated injuries, initial OR times, date of implant removal and Constant-Murley score of the injured and contralateral shoulder were recorded pre- and after implant removal [7]. The active range of motion was measured with a goniometer. Force testing was performed with an Isobex Isometric digital dynamometer (MDS, Oberburg, Switzerland). Impairment of the anterior branch of the axillary nerve was clinically assessed. To exclude operative related impairment, the postoperative follow-up was planned minimally after six weeks. All patients had radiographs of the shoulder in 20° of external and internal rotation as well as axillary view to determine screw length or perforation, assessing plate height, head shaft angulation, or bone-associated complications (e.g. avascular necrosis, non-union) (Fig. 1). Physiotherapy was initialized after initial fracture treatment and was continued after implant removal.

### Implant and surgical technique

In all cases a five-hole Philos®-Plate (Synthes®, Switzerland) allowing angle stable screw fixation in the humerus head and particularly in osteoporotic bone was used. The minimal invasive antero-lateral delta-split approach, splitting the delta muscle at the level of the anterior raphe and avoiding extending the incision for more than 6 cm distal to the cranial tip of the humerus was preferred [8]. Either percutaneous osteosynthesis techniques or the radiolucent aiming device for Philos®-System was used. Non-absorbable sutures were placed through the rotator cuff tendons and knotted onto the plate for firm fixation and reduction of secondary displacement of the tuberosity [8]. The plate was slid underneath the delta muscle on the humerus shaft. Definitive plate fixation in head was performed with minimal four screws according to the surgeon’s choice according to bone quality or fracture pattern.

### Statistics

Data are reported as *n* (%) or n ± SD. The matched numerical outcome was measured using the Wilcoxon test. A *p* value <0.05 was considered significant. IBM SPSS statistics version 22 (Chicago, Illinois, USA) was used for the statistical analyses.

## Results

### Patients

In our observed period, 20 patients met the inclusion criteria and underwent plate removal for persisting shoulder function impairment. The mean age of the patients was 56 years ± 12 (Table 1). The corresponding cohort of all patients with operative treatment for proximal humerus fracture was 59 years ± 13.

**Table 1:** Patients characteristics after proximal humerus fracture and plate removal

Gender		
	Male	8
	Female	12
Age (y ± SD)		56 ± 12
ASA (*n*)		
	1	8
	2	11
	3	1
Dominant (*n*)		
	Yes	5
	Nein	15
Accident (*n*)		
	Ski	8
	Fall at home	4
	Pedestrian	4
	Miscellaneous	4
OR time (min)		
	Fracture fixation	86 ± 26 min
	Implant removal	35 ± 10 min

*SD* standard deviation, *OR* operation room

Radiological analysis showed a correct height of the plates in all cases. In accordance with the operation instructions, the plates were placed 6.9 ± 3 mm distal to the greater tubercle (Fig. 1). The reason for implant removal was subjective restriction in 55 %, pain in 20 %, meteorosensitivity in 15 % and patient wish without any impairment in 10 %.

### Fracture pattern

The dominant arm was injured in 5 (25 %) patients. According to the AO/OTA classification, 60 % of the fractures were classified as type B. Winter sport accidents, i.e. ski and snowboard, accounted for every second fracture (Table 1).

### Follow-up period

The implants were removed after a mean time of 13 ± 5 months. After implant removal, a follow-up appointment was organized after 9 ± 4 weeks.

### Operative procedure

The surgeons required 35 ± 10 min to remove the implant. On a regular basis, blunt separation of the rotator cuff and deltoid muscle was necessary for implant removal. All screws and plates could be removed. Arthroscopy wasn’t performed for implant removal.

### Clinical and functional outcome

**Fig. 2:**
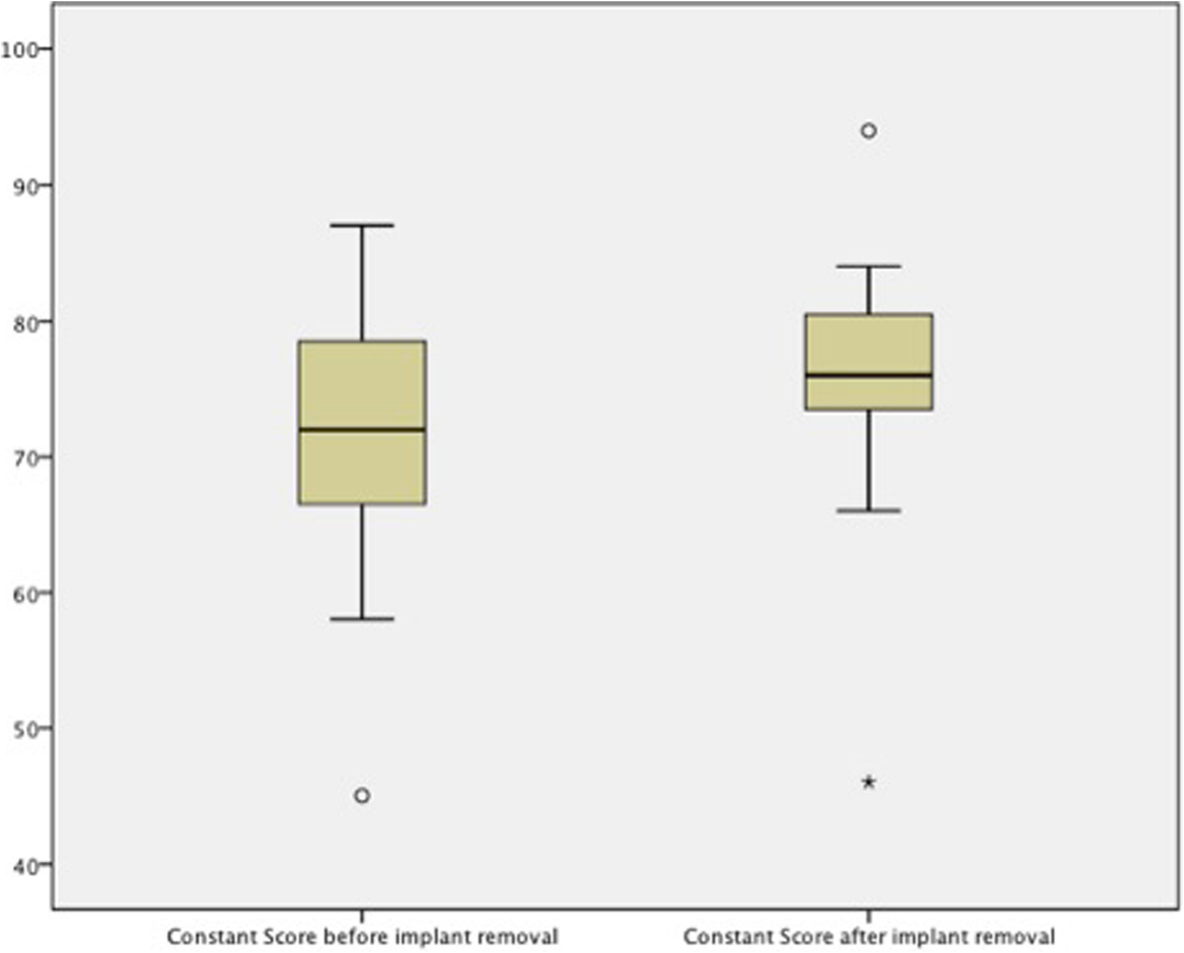
Significant clinical improvements in symptomatic patients after implant removal after proximal humerus fractures

Clinically, the preoperative Constant score was 70.8 ± 9.4. This value increased to 75.6 ± 9.3 after implant removal (*p* = 0.008) (Fig. 2). The 95 % confidence interval of the difference was 1.8–7.8 and the mean difference of the Constant score pre- and postoperative was 4.8 ± 6.4. There was no clinical sign of axillary nerve damage after implant removal.

At the time of preoperative follow-up, avascular necrosis was present in 1 case and screw tip peroration in 3 cases.

## Discussion

Our study presents the results after plate removal in patients with persisting pain or impaired function after proximal humerus fractures. Our patient population showed a significant clinical improvement.

Implant removal uses a substantial part of resources and accounts for up to 30 % of all elective orthopaedic surgical procedures. It is therefore astonishing, that publications addressing complications of implant removal and disadvantages of retained hardware are rare [5]. In a general population, Richards et al. noticed a considerable improvement after implant removal in 91 % of symptomatic patients [9]. But the initial operations included plates, intramedullary nails and tension band wires on multiple body regions.

A number of studies have analysed the functional outcome after locking plate osteosynthesis for displaced fractures of the proximal humerus [3, 4]. Clinical function i.e. Constant Score improved after six to twelve months to 91 % of the contra-lateral uninjured side [4]. After this period, the Constant Score doesn’t further improve significantly as reported by Hirschmann et al. [3]. In several outcome studies, proximal humerus fractures treated with plate osteosynthesis achieved a Constant score from 61 to 75. This value correlates to about 80–90 % of the Constant score of the uninjured shoulder with a score around 80 [3, 4, 10, 11]. We recorded a Constant score in our population of 71 before implant removal. Therefore, a large improvement with continuing conservative and physiotherapy would not have been expected. Meteorosensitivity and impingement might be a reason for the persisting functional impairment and discomfort. It is therefore not surprising, that implant removal is an offered solution to potentially improve function. Lovald et al. assessed the incidence of implant removal after humerus (proximal, shaft and distal) [12]. They found that implant removal was performed in about 10 % of all cases But it seemed, that many of these procedures were associated with the type of health care insurance in the US and age of the patients. Richards et al. confirmed age as a major influential factor for implant removal [9, 12].

In our population, we found a statistical significant improved Constant score of 4.8 points after implant removal. On the first sight, this doesn’t seem to be a huge clinical improvement and it is difficult to quantify the clinical relevance. But the functional analysis of Hirschmann et al. showed a Constant score improvement of only 9.3 during the entire fracture healing, which is know to make a significant clinical difference [3].

Proximal humerus fractures are often accompanied with other shoulder injuries. Visser et al. analysed 143 consecutive fracture of the proximal humerus [13]. They found a temporary axillary nerve injury in 58 % and rotator cuff injury in 11 %. Especially for rotator cuff injuries however, it difficult to differentiate if the tears are caused by the trauma or were pre-existing. The prevalence of asymptomatic rotator cuff tears is age-related and can be found in 20–30 % of the population between 60 and 69 years [14, 15]. We did not do any additional diagnostics e.g. shoulder arthroscopy but accompanying injuries must be considered as a potential source of pain or impaired range of motion.

Major concerns against hardware removals are very high complication rates of 20–47 % [16, 17]. Particularly in locked compression plates, the most frequently observed complication were jammed screws (11 % risk) and damaged recess in which the screwdriver turned freely [17]. A longer time in situ contributed to the complication rate. The relatively short time in situ might have been a reason, why we didn’t observe any removal complications.

But there are some limitations to the study. Since a second operation was only recommended for symptomatic patients, we can only report the results of a small population. But never the less, clinical evaluations showed a significant improvement after implant removal.

## Conclusion

Implant removal seems to statistically significantly improvement of the Constant score after proximal humerus fractures. A more liberal implant removal indication can be considered in patients with persisting pain or decreased range of motion but accompanying shoulder injuries should be excluded.

### Ethical approval

The Cantonal Ethic Committee of Zurich approved the study (KEK-ZH-Nr. 2010-0421/4).

## Abbreviations

AO/OTA: Arbeitsgemeinschaft für Osteosynthesefragen/Orthopaedic Trauma Association
OR: operation room
